# Case Report: The novel hemizygous mutation in the *SSR4* gene caused congenital disorder of glycosylation type iy: A case study and literature review

**DOI:** 10.3389/fgene.2022.955732

**Published:** 2022-10-26

**Authors:** Jun Wang, Xingqing Gou, Xiyi Wang, Jing Zhang, Nan Zhao, Xiaohong Wang

**Affiliations:** Center for Reproductive Medicine, Department of Gynecology and Obsterics, Tangdu Hospital, The Air Force Military Medical University, Xi’an, Shaanxi, China

**Keywords:** *SSR4* gene, congenital disorder of glycosylation, *type Iy*, psychomotor retardation, microcephaly, X-linked *SSR4*-CDG

## Abstract

**Background:** Recently, the hemizygous variation of *SSR4* gene has been reported to be associated with congenital disorder of glycosylation type Iy. To date, only 13 patients have been diagnosed with *SSR4*-CDG in the worldwide, but it has not been reported in the Chinese population.

**Methods:** Whole-exome sequencing and gene copy number variation analysis were used to genetic analysis. The mRNA expression of *SSR4* gene in blood was detected by Real-time Quantitative PCR. The clinical manifestations of all patients reported in the literature were reviewed.

**Results:** WES analysis identified a *de novo* hemizygous variant c.269G>A (p.Trp90*) of *SSR4* gene in the proband with psychomotor retardation, microcephaly, abnormal facial features, and nystagmus. This variant has not been reported in previous studies. The *in vivo* mRNA expression of *SSR4* gene in patient was significantly decreased. Literature review showed that all 14 patients, including our patient, presented with hypotonia, intellectual disability, developmental delay, microcephaly, and abnormal facial features, while most patients had feeding difficulties, growth retardation, and ocular abnormalities, and epilepsy and skeletal abnormalities are less common.

**Conclusion:** We reported the first case of *SSR4*-CDG caused by *SSR4* variant in Chinese population, expanded the clinical and mutation spectra of the disorder, clarified the genetic etiology of the patient, and offered support for the prenatal diagnosis of the index family.

## 1 Introduction

Congenital disorders of glycosylation (CDG) belong to a group of inherited metabolic diseases caused by defects in genes that play an important role in protein and lipid glycosylation. They have high genetic and clinical heterogeneity (Ondruskova et al., 2020). Studies have reported more than 130 glycosylation-related diseases, of which more than 50 diseases involve the N-glycosylation pathway (Freeze et al., 2014), and the *PMM2*-CDG gene mutation is the most reported glycosylation disorder (Wu et al., 2015). Congenital disorder of glycosylation, type Iy (CGDIy, OMIM: 300,934) caused by *SSR4*-CDG gene mutation was first identified in a 16-year-old male patient by Losfeld et al., in 2014 (Losfeld et al., 2014).

According to the professional edition of HGMD, there have only been 13 patients with a definite genetic diagnosis of X-linked *SSR4*-CDG in the world, and most of them have *de novo* mutations. The clinical manifestations including global developmental delay, microcephaly, hypotonia, and intellectual disability, accompanied by facial and ocular abnormalities, with epilepsy and skeletal abnormalities occurring in a small number of patients (Ng et al., 2015). A recent study reported that patients with X-linked *SSR4*-CDG exhibited connective tissue disease, further expanding the phenotypic spectrum of the disease (Castiglioni et al., 2021). This study is the first report of a Chinese patient with X-linked *SSR4*-CDG caused by a nonsense variant of the *SSR4* gene, upon review of the relevant cases reported in previous literature. Our purpose is to improve clinicians’ understanding of the disease, identify the cause of disease in the child from the perspective of genetics, and offer support for the prenatal diagnosis of the pedigree affected with *SSR4*-CDG.

## 2 Materials and methods

### 2.1 Patient clinical information

**FIGURE 1:**
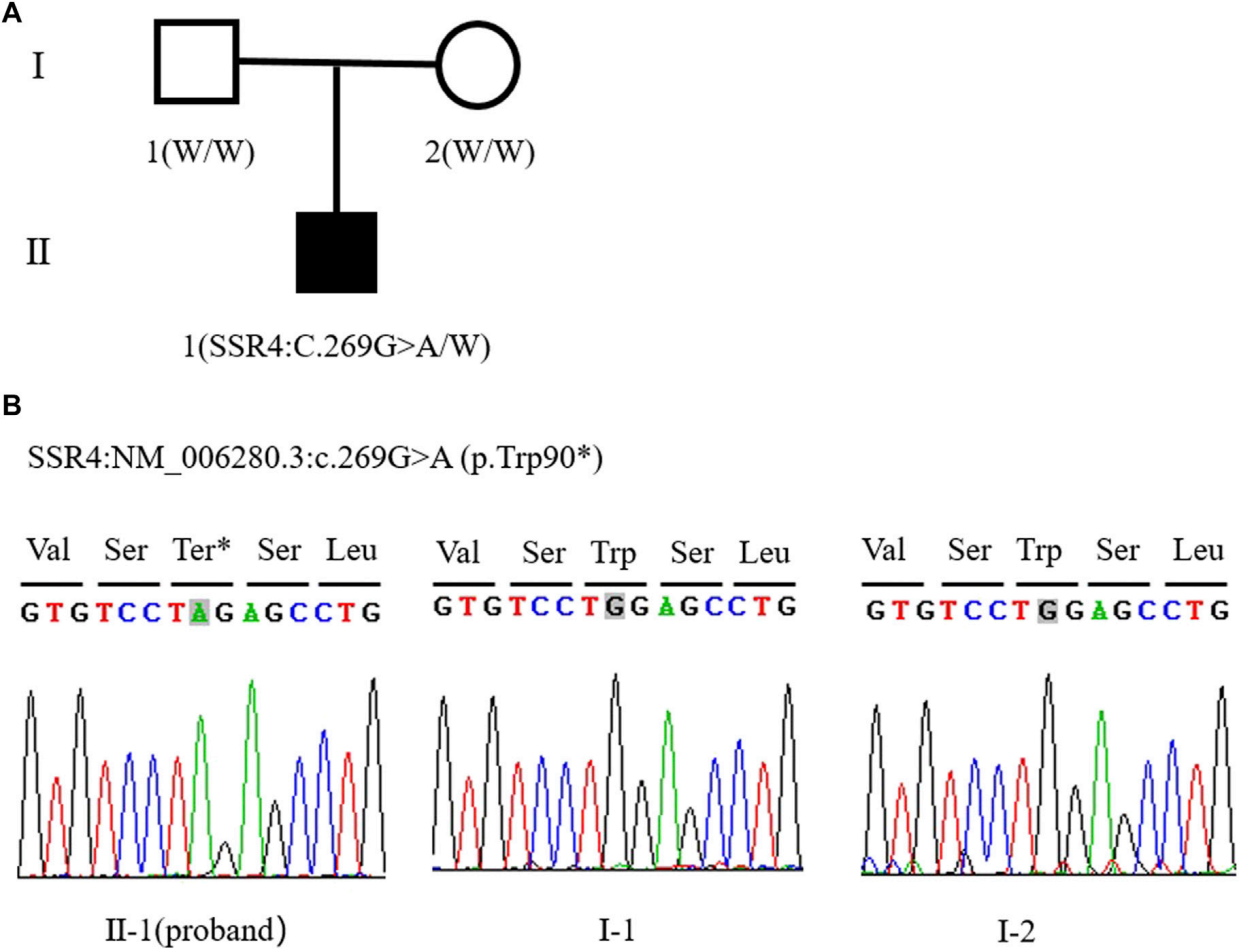
**(A)** Family tree of this study (W: Wild type allele). **(B)** Sanger sequencing peak of SSR4 gene c.269G > A mutation in proband II - one; I-1. The father of the child; I-2. The mother of the child.

The patient was male and had a birth weight of 2,620 g. He was the first child of his parants (G 1P1). His parents were healthy and have no family history (Figure 1A).

At the age of 2 months, he was found to have hypotonia and difficulty in raising his head in the child healthcare department. At the age of 4 months, no disease-related chromosomal copy number variations (CNVs) or loss of heterozygosity were detected by comparing genomic hybridization (CGH) and SNP Array (4 × 180 K). As a type II atrial septal defect was detected on the heart, he was diagnosed as having congenital heart disease. At the same time, he also had retarded psychomotor development and congenital laryngeal cartilage dysplasia. At the age of 5 months and 26 days, we found he had ptosis of his left eye, but neostigmine test was negative and he was diagnosed as having congenital ptosis of the upper eyelid of the left eye, microcephaly, large ears, and a small cleft in the left eye. At the age of 9 months, his brain MRI showed that posterior and anterior of pituitary gland has abnormal signals, and he was further diagnosed with Rathke’s cleft cyst. This patient denied inherited metabolic diseases family history. He had no special body odor, there were no yellowish white skin and hair as well as denied psychomotor retardation and therefore we ruled out phenylketonuria (PKU) temporary. Since the simple physical examination of patient were normal such as daily diet, daily exercise, no abnormal distension and constipation, so we excluded the patient had Congenital Hypothyroidism (CHT). Since birth, he had no feeding difficulty, no development delay, general social interaction like smile well and newborn screening (NBS) for Methylmalonic acidemia (MMA) was normal, accordingly, the patient could be ruled out MMA. At the age of 1 year, In the physical examination, we found the patient had nystagmus and refractive error, and therefore we consulted Ophthalmologist for any relative eye diseases, he was diagnosed with horizontal nystagmus of the left eyeball, bilateral refractive errors, neck softness, and hypotonia. The patient’s result of carbohydrate deficient transferrin (CDT) test (nephelometry) was abnormal elevated, and parental normal test results. At the age of 3 years, his parents planned to have another child and came to our reproductive medical center. Since the etiology of the child was unclear, whole exome sequencing (Trio-WES) of the pedigree, combined with a medical history and pedigree investigation, was recommended. According to genetic counseling, the parents were informed that it was a *de novo* variant, the chance of reoccurrence in next pregnancy was low. Natural pregnancy can be attempted. However, considering the possibility of gonad mosaicism, prenatal diagnosis is necessary in the second trimester. After careful consideration, both couples accept the plan of natural pregnancy and prenatal diagnosis in the second trimester.

This study was approved by the Ethics Committee of the Tangdu hospital of the Air Force Military Medical University, China (K202201-01). And an informed consent form was signed by the parents of the patient.

### 2.2 Whole exome sequencing analysis

EDTA anticoagulant tubes were used to extract 4 ml of peripheral blood from the child and his parents, respectively, and the child’s whole genome DNA was extracted using QIAamp DNA Blood Mini Kit (the operation was performed according to the instructions) and stored at -2 °C for later use.

A NanoWES probe was used for hybrid capture of the whole exome DNA of the pedigree. Other tools used were the Nova Seq 6,000 platform for high-throughput sequencing, an hg38 human genome assembly for comparison, and the Verita Trekker variant site detection system and Enliven variant site annotation interpretation system for data analysis. Mutation sites with mutation frequency greater than 1% (according to 1,000 Genomes, ExAC, gnomAD, and other databases), as well as non-functional mutation sites (such as synonymous mutation and mutation in non-coding regions), were removed. After the pathogenicity predictions were conducted in SIFT, Polyphen2, CADD, and other software, candidate gene mutation sites were screened according to clinical symptoms, related disease database queries, and literature references, and Sanger sequencing was used for pedigree verification. The primer sequences are as follows: *SSR4*-4F:CCAGAACATGGCTCTCTATGCT. *SSR4*-4R: GGG​GAA​AGA​CAG​GTA​GGA​ACA​C. The pathogenicity rating of mutation sites and data interpretation rules refer to the guidelines of the American College of Medical Genetics and Genomics (ACMG) and the recommendations of the ClinGen Sequence Variant Interpretation (SVI) Working Group on the application of the guidelines (Richards et al., 2015).

### 2.3 RNA extraction and quantitative RT-PCR assays

2.5 ml whole blood was transferred to the blood RNA preservation tube (BioTeke (Beijing) Co., Ltd., No. st1001), fully lysed and extracted blood RNA by the blood RNA Extraction Kit (BioTeke (Beijing) Co., Ltd., No. rp4001). 1ug RNA was used to reverse transcribe into cDNA by the reverse transcription Kit (Yeasen Biotechnology (Shanghai) Co., Ltd., No. 11123es70). Two primers for QPCR were designed using Primer five software:

*SSR4*-1F: CCC​AGA​TCA​CCC​CTT​CCT​AC.

*SSR4*-1R: CCT​CGA​GTG​ACA​GGG​AAT​TG.

*SSR4*-2F: CTC​AGG​AAG​GCT​CAG​AGG​AA.

**FIGURE 2:**
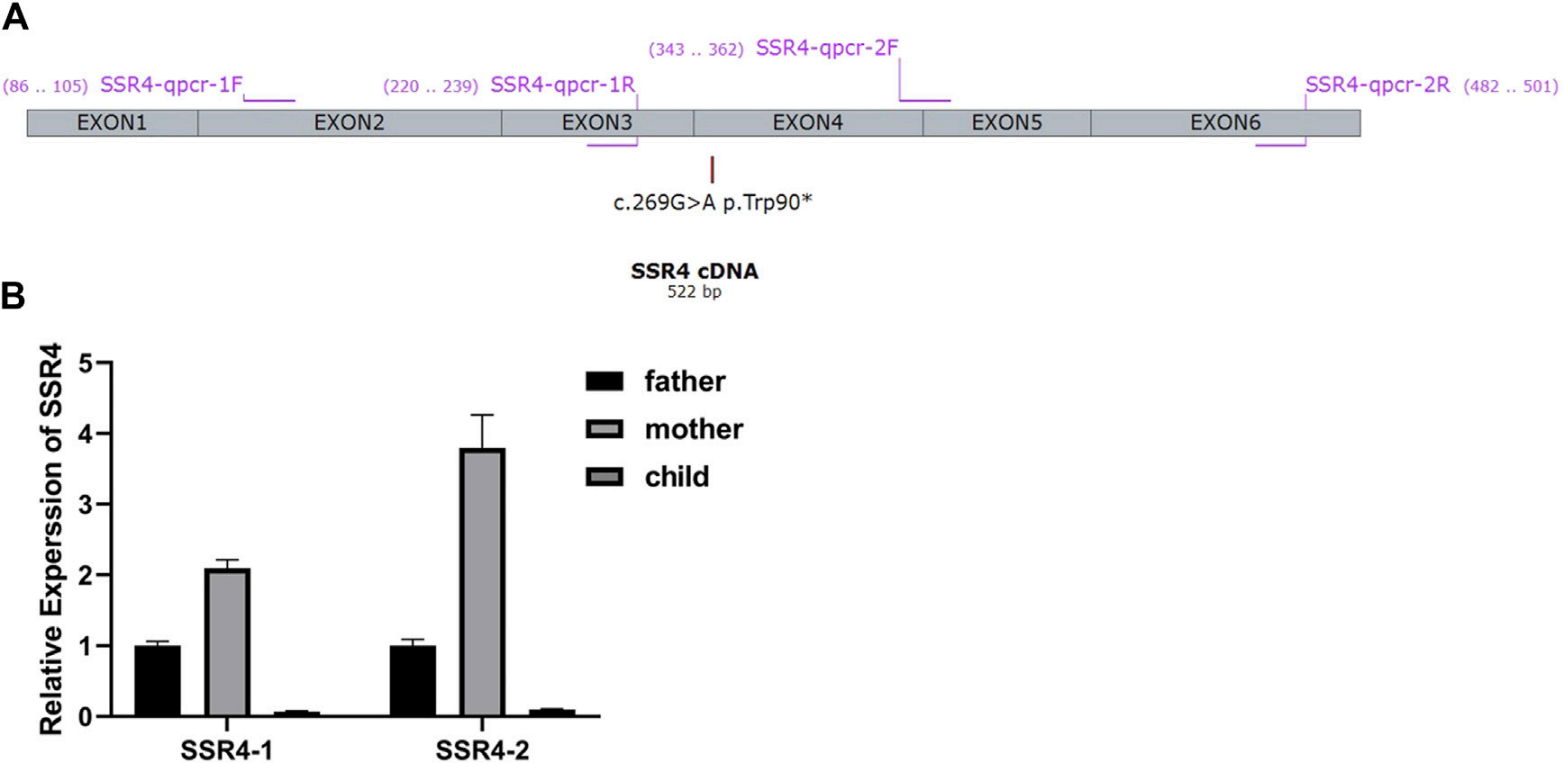
**(A)** Primer design for qPCR According to the gene sequences upstream and downstream of the mutation site, two pairs of primers were designed using Primer five software. Primers specifically amplify the target region of the SSR4 gene. The length of the amplified product is in the range of 100-200bp. The product cannot form secondary structures. **(** B **)** mRNA expression. Expression levels were analyzed with normal males (father of the proband) as controls.

*SSR4*-2R: CGC​ACT​GAA​GGC​CAA​GTA​GT.

(Bio-Rad, CFX connect real time system) (Figure 2A). Result of QPCR was determined by SYBR Green Master Mix (Yeasen Biotechnology (Shanghai) Co., Ltd., No.11201es03). All experiments of the three samples were repeated three times, and the Ct values corresponding to each reaction conditions were averaged. All experiments of the three samples were repeated three times, and the Ct values corresponding to each reaction conditions were averaged. Actin gene was using as internal reference. The proband’s father was selected as a normal control. The relative expression level was calculated by the comparative CT method (ΔΔCT).

## 3 Results

### 3.1 Gene analysis results

Trio-WES showed that the patient carried hemizygous variant NM_006280.3:c.269G>A (p.Trp90*) in the *SSR4* gene (chrX:153797732, hg38), his mother did not carry the variant, so the variant was confirmed as *de novo.* But the possibility that his mother carries germ cell mosaicism cannot be ruled out. The Sanger sequencing verification results were consistent with the Trio-WES (Figure 1B). p. Trp90* variant has not been included in the Exome Aggregation Consortium (ExAC), 1,000 Genomes (1000G), or Genome Aggregation Database (gnomAD). This nonsense variant p. Trp90* may produce a premature termination of protein. According to ACMG guidelines, this variant is classified as pathogenic (PVS1+PS2+PM2-Supporting). The data filtering principles are shown in Supplementary Table S1.

### 3.2 mRNA analysis of patient sample

Two pairs of primers SSR4-1-F/R (Upstream of variation locus) and SSR4-2-F/R (downstream of variation locus) were designed to detect the mRNA expression of the patient. 2^–∆∆Ct^ method was used to calculate the relative fold gene expression of samples when performing real-time polymerase chain reaction. With the expression level of father as control, SSR4-1 F/R primers test results showed that the expression level of mother was 2.09, and the expression level of patient was 0.07. SSR4-2F/R primer test results showed the expression level of the mother was 3.80 and that of the patient was 0.10 (Figure 2B). It indicated there had mRNA degradation in the patient’s sample, which may result from nonsense-mediated mRNA decay (NMD).

### 3.3 Literature search results

**FIGURE 3:**
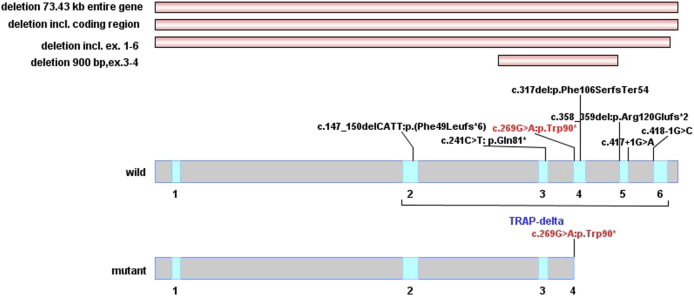
Distribution of SSR4 gene mutation. (The red horizontal stripes are the previously reported type of copy number variations. In wild-type and mutant protein domains, the red font is the variation reported in this study, and the black variations are previously reported. The blue area of delta subunit precursor (TRAP-delta) contains five exons from exon2 to exon6).

“Congenital disorder of glycosylation, type Iy”, “congenital deglycosylation, type Iy” and “*SSR4* gene” were used as keywords to search in the China National Knowledge Infrastructure (CNKI), China Online Journals-Wanfang Data Knowledge Service Platform, and Chinese VIP Database (up to December 2021), and no case reports related to CGDIy were retrieved. “*SSR4*″ and “Congenital disorder of glycosylation, type Iy” were used as the keywords to search in the PubMed database (up to December 2021), and a total of four papers related to *SSR4* gene mutation involving 13 patients were retrieved (Losfeld et al., 2014;; Ng et al., 2015; Medrano et al., 2019; Castiglioni et al., 2021). All 14 patients (including the patient in this paper) are male, with clinical manifestations of developmental delay (14/14), intellectual disability (14/14), microcephaly (14/14), hypotonia (14/14), and facial abnormality (14/14). Among them, facial abnormalities mainly include a small jaw, large ears, sunken eye sockets, a large mouth, large spacing between teeth, and strabismus. Most patients have ocular abnormalities (11/14), feeding difficulties (10/14), gastrointestinal problems (10/14) in infancy, as well as postnatal growth retardation (10/14). Table 1 was summary of the clinical findings and the variants in the 14 children with X-linked *SSR4*-CDG. Figure 3 was drawn using IBS software according to the instruction (Liu et al., 2015).

**TABLE 1:** The clinical findings and the variants in the 14 children with X-linked SSR4-CDG.

Patients	1	2	3	4	5	6	7	8	9	10	11	12	13	14	Cases
Sex	Male	Male	Male	Male	Male	Male	Male	Male	Male	Male	Male	Male	Male	Male	
Age/Origin	10years/Asian	4years/Brazilian	2years/Brazilian	4years/European	14years/Hispanic	13years/Hispanic	5years/Hispanic	NA/Hispanic	16 years/NA	16 years/NA	12 years/French	2 years 2 m/Chilean	2 y10 m/French	3years/Chinese	
Age at diagnosis	NA	NA	NA	NA	NA	NA	NA	NA	NA	NA	8 years	9 months	6 months	3years	
Variant	g.153062612_153063511del	c.358_359del, p.Arg120Glufs*2	c.358_359del, p.Arg120Glufs*2	g.153031975_153105401del	c.417 + 1G>A	c.418-1G>C	c.418-1G>C	c.418-1G>C	c.317del, p.Phe106SerfsTer54	c.147_150del, p. (Phe49Leufs*6)	c.241C>T, p.Gln81*	arr [GRCh37]Xq28 (153011909_153063825)x0	arr [GRCh37]Xq28 (153060022_153063888)x0	c.269G>A p.Trp90*	
Inheritance	*de novo*	Gonadal mosaicism	Gonadal mosaicism	maternal	*de novo*	maternal	maternal	maternal	*de novo*	*de novo*	*de novo*	*de novo*	maternal	*de novo*	
Developmental retardation	+	+	+	+	+	+	+	+	+	+	+	+	+	+	14/14
Mental retardation	+	+	+	+	+	+	+	+	+	+	+	+	+	+	14/14
Microcephaly	+	+	+	+	+	+	+	+	+	+	+	+	+	+	14/14
Abnormal facial shape	+	+	+	+	+	+	+	+	+	+	+	+	+	+	14/14
Muscular hypotonia	+	+	+	+	+	+	+	+	+	+	+	+	+	+	14/14
Feeding difficulties	+	+	+	+	+	+	+	-	+	-	+	+	-	-	10/14
Abnormality of the gastrointestinal tract	+	+	+	+	+	+	+	-	Gastroesophageal reflux	-	-	Gastroesophageal reflux, gastrostomy, difficulties/aversion to food with certain textures	Gastroesophageal reflux, no dysphagia	-	10/14
Growth retardation	+	+	+	+	-	+	+	-	+	-	+	+	+	-	10/14
Visual acuity test abnormality	-	+	+	+	+	+	+	+	-	+	+	-	+	+	11/14
Brain MRI	-	-	-	+	-	+	+	-	-	+	+	+	+	-	7/14
Skeletal Anomalies	-	-	+	+	+	-	-	-	-	-	Joint laxity, severe deformation of the feet in valgus, orthopedic surgery (11.5 years)	Fifth finger clinodactyly, joint laxity	Superposition of the second toe without syndactyly, joint laxity	-	6/14
Epilepsy	+	-	-	+	+	-	-	+	Seizure disorder was mild	Febrile	-	-	-	-	6/14
Abnormality of coagulation	+	-	-	-	-	-	-	-	Persistent bleeding and easy bruising	-	FVIII = 48% [60–150%]; vWF 34% [50–150%]	APTT = 11.2 [24.8–33.2 s]; PT = 97% [80–120%]	-	-	4/14
Cardiac anomalies	-	-	-	-	+	-	-	-	-	-	-	-	-	Congenital type II Atrial septal defect	2/14
Abnormality of the kidney	-	-	-	-	+	-	-	-	-	-	-	-	-	-	1/14
References	Ng et al. (2015)	Losfeld et al. (2014)	Medrano et al. (2019)	Castiglioni et al. (2021)	This work										

Transcript, NM_006280.3. y, year. m, month.

## 4 Discussion

Glycosylation is one of the most common post-translational modifications of proteins and lipids, and it plays a crucial role in the growth and development of organisms (Moremen et al., 2012). -Congenital disorders of glycosylation (CDG) are a series of metabolic disorders due to defects in a complex chemical process known as glycosylation. Glycosylation involves multiple biosynthetic pathways, mainly N-glycosylation, which widely affects various systems and organs. Therefore, affected individuals have complex and diverse clinical phenotypes and are difficult to diagnose (Freeze et al., 2014; Ferreira et al., 2018; Ng and Freeze, 2018). The clinical phenotype varies depending on the organ involved, with the brain, eyes, and bones generally being the most commonly affected (Francisco et al., 2019). The clinical manifestations are predominantly neurological, including psychomotor retardation or cognitive disorders, epilepsy, hypotonia, ataxia, polyneuropathy, and stroke-like events (Freeze et al., 2015; Gardeitchik et al., 2018; Paprocka et al., 2021). Some patients also have deformities, such as facial deformity, inverted nipples, and subcutaneous fat pads (Francisco et al., 2019).

The modes of inheritance of CDG include autosomal recessive inheritance, autosomal dominant inheritance, and X-linked recessive inheritance. The *SSR4*-CDG mutation reported in this study can lead to an X-linked recessive congenital disorder of glycosylation, type Iy (OMIM: 300,934). As of February 2021, 13 patients with X-linked *SSR4*-CDG have been included in the professional edition of HGMD. The 13 patients have five common clinical phenotypes, including psychomotor retardation, intellectual disability, facial abnormality, microcephaly, and hypotonia. Most patients (10/13) have gastrointestinal problems caused by feeding difficulties, growth retardation, and visual problems such as nystagmus and refractive errors. Half of the patients (6/13) have non-specific imaging abnormalities, including delayed myelination, corpus callosum dysplasia, decreased ventricular white matter, and absence of transparent septum. More than half of the patients have epilepsy or febrile seizures and skeletal abnormalities (skeletal deformities, scoliosis, and delayed bone age). A minority of patients have coagulation disorders, cardiac abnormalities (2/13), and renal abnormalities (1/13) (Castiglioni et al., 2021). The clinical features of the present patient are essentially consistent with the phenotypes of the X-linked *SSR4*-CDG patients reported in previous literature. In addition to the five typical features, he also showed obvious vision problems, nystagmus, bilateral refractive errors, cardiac abnormalities, and special features such as high arched eyebrows and low ears. However, it is worth noting that these typical manifestations are not only limited to *SSR4* gene-related CDG patients, but also may be associated with other CDG subtypes, such as congenital disorders of glycosylation caused by *SSR3* gene variants or syndromes associated with developmental delay. For similar clinic manifestations patients, phenotypes overlap each other. Therefore, simple and economical biochemical tests, such as the determination of carbohydrate deficient transferrin (CDT), can be performed firstly, then followed by further genetic testing for accurate molecular typing. Due to the high clinical and genetic heterogeneity of glycosylation disorders, it is difficult to diagnose based on clinical manifestations alone, so genetic testing and analysis should be performed as soon as possible.

Translocon-associated protein (TRAP) is a membrane protein that is ubiquitous in all eukaryotes (Sommer et al., 2013). It is located in the endoplasmic reticulum membrane as a signal sequence receptor protein and is involved in protein transport across the endoplasmic reticulum membrane (Braunger et al., 2018). The TRAP complex consists of four signal sequence receptor proteins (SSR1-4), of which the *SSR4* gene encodes the delta subunit (Hartmann et al., 1993). Nagasawa et al. have confirmed that knocking out any subunit of the TRAP complex will affect the function of the entire complex (Nagasawa et al., 2007), while another study has shown that the TRAP complex binds to the oligosaccharide transferase complex and directly participates in N-glycosylation. Additionally, overexpression of the wild-type *SSR4* allele can restore the glycosylation of other members of the TRAP complex (Losfeld et al., 2014).

A recent study identified the TRAP complex as a key regulator for the maintenance of protein glycosylation modifications under conditions of cellular stress (Phoomak et al., 2021). The *SSR4* gene (NM_006280.3) consists of six exons encoding 173 amino acid residues, of which amino acids 1–23 are the signal peptide sequences of the protein and amino acids 23–173 constitute the important functional domain structure of the *SSR4* protein TRAP-δ subunit. The p. Trp90* mutation of the *SSR4* gene found in this study is located in exon 4 (Figure 3), which can cause amino acids after the 90th position of the *SSR4* protein to be unable to be translated normally. As a result, a truncated protein with only 90 amino acids is produced, the spatial structure of the protein is destroyed, and the TRAP-δ subunit becomes incomplete; which, in turn, affects the normal function of the *SSR4* subunit and is expected to affect the role of TRAP complex in endoplasmic reticulum protein transport and glycosylation modification.

A total of 10 *SSR4* gene mutations associated with CDG have been included in PubMed and HGMD databases so far, including one nonsense mutation, two splicing mutations, three frameshift mutations caused by deletions, and four large fragment deletions, with no missense mutations having been reported. Seven of these mutations are *de novo* variants, and three (c.418-1G>C, 73.43 kb deletion, and deletion in the Xp28:153060022–153063888 region) are inherited from mothers with mild symptoms or from asymptomatic mothers, respectively. As a haploinsufficiency of glycosylation disorder causative gene, all the reported pathogenic mutations in *SSR4* gene were loss-of-function, four of which were located in the downstream region of c.269G>A. It’s predicted that they can produce shorter truncated protein and induce NMD mechanism. In this study, by detecting the mRNA expression of the *SSR4* gene in the blood, the mRNA expression of *SSR4* gene in the blood of the patient was significantly reduced than that of normal controls, which might be due to the degradation of mRNA caused by NMD. This may result in impaired protein function and lead to disease.

All the mutations are loss-of-function and there is no report on the pathogenicity of missense mutations, which may be related to the pathogenesis of the disease itself. Correlations between genotype and phenotype are difficult to establish due to the limited reported genetic mutations and few cases. This paper reports the first case of X-linked *SSR4*-CDG in China, enriches the mutation and phenotype spectra of the *SSR4* gene, improves clinicians’ understanding of the disease, provides reliable genetic evidence for prenatal diagnosis of the pedigree, and lays a foundation for the follow-up study of the molecular mechanism of the disease.

At present, the whole-exome sequencing technology has been widely used in the detection of clinical genetic diseases. As the sequencing technology becomes mature and the costs reduce, more genetic diseases will be discovered, especially in children with psychomotor retardation. Genetic testing not only provides precise directions for the diagnosis and treatment of children with rare diseases but also prevents birth defects and promotes high-quality development. At present, most of the treatments for children with genetic diseases are symptomatic treatment and management. Early detection and early intervention can reduce the occurrence of complications and improve children’s quality of life.

## Data Availability

The original contributions presented in the study are included in the article/Supplementary Material, further inquiries can be directed to the corresponding author.
